# Deciphering the tRNA-derived small RNAs: origin, development, and future

**DOI:** 10.1038/s41419-021-04472-3

**Published:** 2021-12-21

**Authors:** Bowen Liu, Jinling Cao, Xiangyun Wang, Chunlei Guo, Yunxia Liu, Tianjiao Wang

**Affiliations:** 1 Research Center for Molecular Oncology and Functional Nucleic Acids, School of Laboratory Medicine, Xinxiang Medical University, 453003 Xinxiang, Henan PR China; 2 Department of Medicine, University of Wisconsin-Madison, Madison, WI 53705 USA; 3 State Key Laboratory of Medicinal Chemical Biology, Department of Biochemistry, College of Life Sciences, Nankai University, 300071 Tianjin, PR China

**Keywords:** Oncogenes, Non-coding RNAs

## Abstract

Transfer RNA (tRNA)-derived small RNAs (tsRNAs), a novel category of small noncoding RNAs, are enzymatically cleaved from tRNAs. Previous reports have shed some light on the roles of tsRNAs in the development of human diseases. However, our knowledge about tsRNAs is still relatively lacking. In this paper, we review the biogenesis, classification, subcellular localization as well as action mechanism of tsRNAs, and discuss the association between chemical modifications of tRNAs and the production and functions of tsRNAs. Furthermore, using immunity, metabolism, and malignancy as examples, we summarize the molecular mechanisms of tsRNAs in diseases and evaluate the potential of tsRNAs as new biomarkers and therapeutic targets. At the same time, we compile and introduce several resource databases that are currently publicly available for analyzing tsRNAs. Finally, we discuss the challenges associated with research in this field and future directions.

## Facts

- tsRNAs are produced by tRNA cleavage and can regulate gene expressions at transcriptional and translational levels.
- Chemical modifications of tRNAs can directly affect the production and biological functions of tsRNAs.
- tsRNAs are involved in the pathogenesis of multiple diseases including immune disorders, metabolic disorders, and in the development of malignant tumors, but the detailed molecular mechanism is yet to be elucidated.
- tsRNAs are potential diagnostic biomarkers and therapeutic targets for various diseases.

## Open questions

- How are tsRNAs degraded?
- During their functions, do tsRNAs interact with other noncoding RNAs?
- What are the chemical modifications on tsRNAs, and do these modifications affect their biological functions?

## Introduction

The past decade has changed our understanding of noncoding RNAs (ncRNAs), from unappreciated junk transcription products to important functional molecules involved in cellular processes [1]. For instance, long noncoding RNAs (lncRNAs), which are usually longer than 200 nucleotides (nt) [2], regulate gene expressions at transcriptional and translational levels by interacting with mRNA, DNA, proteins, and other ncRNAs [3–6]. Circular RNAs (circRNAs) are a group of covalently closed single-stranded transcripts that influence the translation processes of other transcripts through sponge actions [7–9]. Small noncoding RNAs (sncRNAs), which range from 18~200 nt in length, are widely present inside and outside cells [10, 11]. SncRNAs can be classified into several subtypes, including microRNAs (miRNAs), small interfering RNAs (siRNAs), small nuclear RNAs (snRNAs), and PIWI-interacting RNAs (piRNAs) among others [12, 13]. They regulate basic biological processes such as cell growth, differentiation, and play roles in the development of several human diseases, including nervous disorders, immune-related diseases, and malignant tumors [14–16].

Due to advances in high-throughput technologies, many different sources of sncRNAs have been identified. Ribosomal RNAs (rRNAs)-derived RNA fragments (rRFs), a new class of sncRNAs, have been found to stably exist and function in cells [17, 18]. Small nucleolar RNAs (snoRNAs) have been shown to direct the chemical modifications of rRNAs [19]. Moreover, snoRNAs-derived fragments (sdRNAs) can modulate gene expressions and participate in disease progression [20, 21]. Currently, through research, some neglected transfer RNA (tRNA)-derived small RNAs (tsRNAs) have been gradually discovered.

The main role of tRNAs is to carry amino acids into ribosomes for protein synthesis under the guidance of mRNA. During biogenesis, tRNAs can be specifically cleaved into many small fragments by several enzymes. tsRNAs are mainly classified into tRNA-derived fragments (tRFs) and tRNA halves. However, the significance of these small RNAs has not been fully established.

This review introduces the classification, occurrence, subcellular localization, and action mechanism of tsRNAs, and focuses on the association of chemical modification of tRNA and tsRNAs production. Furthermore, we summarize the representative mechanisms of tsRNAs in disease and evaluate their potential as novel biomarkers and therapeutic targets in disease. In the end, we discuss the existing problems in this field and look into the future development directions.

## Biogenesis and classification of tsRNAs

tRNAs, composed of 70–90 nucleotides, are a class of RNAs with L-shaped tertiary structures and “clover”-shaped secondary structures that are capable of carrying and transporting amino acids [22]. During biogenesis, tRNAs are transcribed into precursor tRNAs (pre-tRNAs) via the action of RNA polymerase III. Then, pre-tRNAs are transformed into mature tRNAs through further processing and modification [22, 23], including removal of the 5’ precursor by endonuclease P (RNaseP), removal of the 3’ trailer sequences by endonuclease Z (RNaseZ)/cytoplasmic homologous ribonuclease Z2 (ELAC2) (resulting in the production of tRF-1); then, in the presence of tRNA nucleotidyl transferase, the trinucleotide “CCA” sequence is attached to the 3’ end of tRNA [24, 25].

**Fig. 1:**
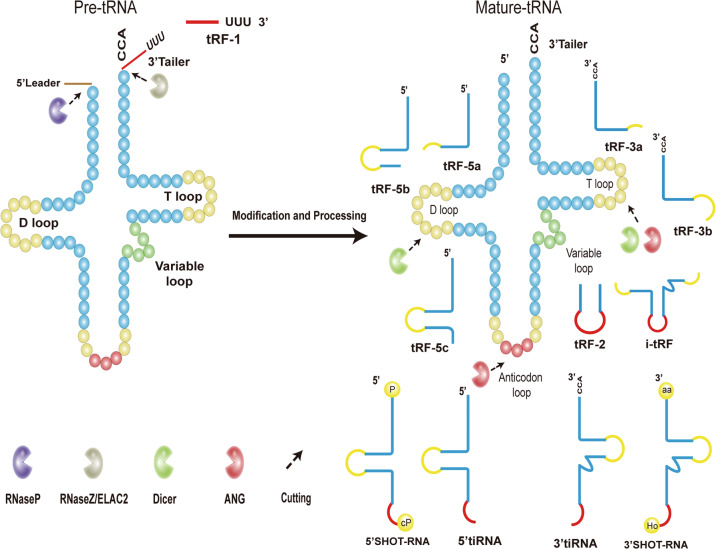
Biogenesis and classification of tsRNAs. tRF-1 is produced by RNaseZ/ELAC2 cleavage of the 3’ trailer sequence of the precursor tRNA. tRF-2 contains the stem sequence and the anticodon loop portion of the mature tRNA. tRF-3 is the 3’ end fragment of tRNA produced by cleavage of the mature tRNA T-loop by nucleases. tRF-5 is produced by Dicer enzyme cleavage of the D-loop of mature tRNA. 5’ tiRNA and 3’ tiRNA are 5’ and 3’ fragments of mature tRNA produced by cleavage from the anticodon loop, respectively. The 5’-SHOT-RNAs are half of the 5’ end of the tRNA with phosphate at the 5’ end and a cyclic phosphate (cP) at the 3’ end. 3’-SHOT-RNAs contain half of the 3’ tRNA with a hydroxyl group at the 5’ end and an amino acid at the 3’ end. The i-tRF is a fragment containing the tRNA anticodon loop and the D- and T-loops.

tRNA halves, which are also termed tRNA-derived stress-inducible RNAs (tiRNAs), are produced by ANG cleaving at the middle of the anticodon loop of mature tRNAs. In most cases, the production of tiRNAs is triggered by stress stimulation, including heat shock, cold shock, hypoxia, and oxidative stress [26–28]. However, tiRNAs, which are composed of 31–40 nucleotides, are also produced and are present in normal cells [29–31]. Based on the inclusion of 5’ sequences or 3’ sequences of the anticodon, tiRNAs can be divided into two subclasses: 5’ tiRNA and 3’ tiRNA [29, 30]. The 5’ tiRNA contains the portion from the anticodon loop cleavage site to the mature 5’ end of the tRNA, while the 3’ tiRNA refers to the anticodon loop cleavage position to the 3′ end (Fig. 1). Honda S et al. reported a group of tiRNAs that were produced under sex hormone stimulation. They are referred to as sex hormone-dependent tRNA-derived RNAs (SHOT-RNAs) [32]. The SHOT-RNAs are produced under the stimulation of estrogen or androgen [32]. They can be divided into 5’-SHOT-RNAs and 3’-SHOT-RNAs. 5’-SHOT-RNAs contain 5’ tRNA half with phosphate at the 5’ end and a cyclic phosphate (cP) at the 3’ end. 3’-SHOT-RNAs contain 3’ tRNA halves with a hydroxyl group at the 5’ end and an amino acid at the 3’ end (Fig. 1) [32].

Broadly, tRF can be classified as: tRF-1, tRF-2, tRF-3, tRF-5, and i-tRF (Fig. 1). tRF-1 is a small fragment generated as a result of RnaseZ/ELAC2 cleavage of the 3′ end of precursor tRNA, a 3’ trailer sequence containing a poly-U sequence [33]. tRF-2 is enzymatically cleaved from the anticodon loop of tRNA-Tyr, tRNA-Gly, tRNA-Asp, and tRNA-Glu, containing the stem sequence and anticodon loop, but lacking the 5′ and 3′ ends of tRNA [33, 34]. tRF-3, which contains 18–22 nucleotides, is the 3’ terminal portion containing the CCA sequence produced by cleavage in the T-loop of mature tRNA by ANG, Dicer, or members of the ribonuclease family. It can be divided into two subclasses: tRF-3a (-18nt) and tRF-3b (-22nt) [35, 36]. tRF-5, a derivative fragment that is less than 30 nucleotides, is produced by the Dicer enzyme acting on the D-loop of mature tRNA. It contains the 5’ end of tRNA. Based on cleavage positions of the Dicer at the D-loop, tRF-5 can be divided into three subtypes: tRF-5a (14–16 nt), tRF-5b (22–24 nt) and tRF-5c (28–30 nt) [33, 37]. i-tRF is cleaved from the mature tRNA region and contains the anticodon loop and the fragments of the D- and T-loops [36, 38, 39]. Currently, there is no uniformity with regards to the naming rules of tsRNAs.

## Subcellular localization of tsRNAs

tsRNAs are mainly localized in the cytoplasm [40, 41]. tRFs are produced by cleavage of mature tRNAs in the cytoplasm by Dicer. At the same time, under stress conditions, ANG translocates from the nucleus and accumulates in the cytoplasm, slicing mature tRNAs to produce tiRNAs [42]. As a special tRF, there are two possible situations with regards to tRF-1 localization. tRF-1 is cleaved from pre-tRNA by RNaseZ in the nucleus, after which it is translocated to the cytoplasm [41]. In another case, tRF-1 may be directly generated in the cytoplasm by RNaseZ from the cytoplasmic pool [43, 44].

Notably, the mitochondria contain 22 tRNA-encoding genes [45], and processing of 3’ ends of tRNAs occurs in the cytoplasm and mitochondria. Moreover, even though ANG is not localized in the mitochondria [46], Dicer and AGO, the two proteins that contribute significantly to tsRNA production and function, have been detected in the mitochondria [47]. These evidence imply that tsRNAs are very likely produced and exist in the mitochondria. In recent years, researchers have obtained the information of tsRNAs in mitochondria [45, 48]. The tsRNAs that are localized in the mitochondria of various tissues can be named mt-tsRNAs [47–49]. Even though the molecular mechanisms of mt-tsRNA biogenesis have not been established, mutations in the mt-tRNA gene and modification status of mt-tRNA have been shown to influence mt-tsRNA production [50].

Meanwhile, the potential association between cytoplasmic tsRNA and mt-tsRNA should be evaluated further. With the help of the mitochondrial intermembrane protein polynucleotide phosphorylase (PNPASE) [51], small RNAs are capable of being imported into the mitochondria, implying the possibility that tsRNAs can be translocated from the cytoplasm into the mitochondria. Interestingly, tsRNAs in the cytoplasm can also be derived from mt-tRNAs. Studies suggest that mt-tRNA might have the ability to be transported from the mitochondria into the cytoplasm, although the mechanism of transport has not been established, the mt-tRNA has been detected in the cytoplasm [52]. Subsequently, in the cytoplasm, mt-tRNAs can be cleaved by Dicer to produce tsRNAs [45]. However, the functions and transport mechanisms of mt-tsRNA should be investigated further.

## Regulation of tsRNA production

A central feature of epitranscriptomics is the chemical modification of RNAs, especially in tRNAs and rRNAs, where base modifications are prevalent [53–55]. Various modifications on tRNAs not only exert effects on tRNA functions, they also shape the fate of tsRNA production.

**Fig. 2:**
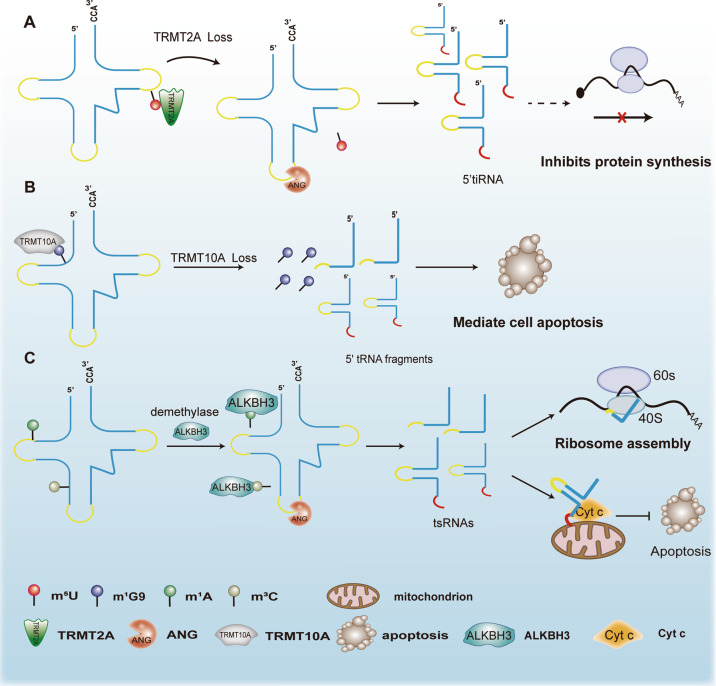
Regulation of tsRNA production. **A** Deletion of the methyltransferase, TRMT2A, induces ANG-dependent production of 5’tiRNA and inhibits the protein synthesis. **B** The lack of the TRMT10A methyltransferase leads to hypomethylated tRNA cleavage to produce a 5’ tRNA fragment that mediates cell apoptosis. **C** In the presence of the ALKBH3 demethylase, tRNA hypomethylation produces tsRNAs that can bind to the 40s ribosome to facilitate ribosomal assembly and interact with cytochrome *c* to inhibit apoptosis, respectively.

At the post-transcription level, methylation is a common chemical modification of tRNA [56, 57]. Blanco S et al. demonstrated that the loss of the methyltransferase, NSun2, directly leaded to tRNA hypomethylation [58]. Hypomethylated tRNA has a high affinity for ANG, which cleaved it, leading to the accumulation of 5’ tRNA fragments. The accumulated 5’ tRNAs were shown to be able to activate the uncapped translational repressor program [58, 59], resulting in reduced protein synthesis [58]. As a tRNA methyltransferase, TRMT2A can catalyze 5-methyluridine (m^5^U) modification at position 54 of cytoplasmic tRNAs [60]. TRMT2A knockdown promoted m^5^U54 tRNA hypomodification and enhanced ANG-dependent production of 5’tiRNA-GlyGCC and 5’tiRNA-GluCTC. Furthermore, Pereira M et al. found that silencing TRMT2A inhibited global protein synthesis, however, they did not establish whether TRMT2A-induced 5’ tiRNA can directly modulate protein synthesis (Fig. 2A) [60]. In pancreatic β-cells, the methylation transferase, TRMT10A, is responsible for methylating guanosine at the tRNA-Gln and tRNA^iniMeth^ position 9 (m^1^G9) [61–63]. The loss of TRMT10A function is a monogenic cause of early diabetes and microcephaly [61]. Cosentino et al. documented that in pancreatic β-cells, deficiencies of TRMT10A resulted in reduced tRNA guanosine methylation. The hypomethylated tRNAs divided, and produced 5’ tRNA fragments (5’ tiRNAs and 5’ tRFs) to mediate β-cell apoptosis (Fig. 2B) [61].

The demethylation activities of ALKBH3, which belongs to the AlkB family and catalyzes biological oxidation using nonheme iron (II), involve DNA and RNA. However, previous studies have mainly focused on DNA demethylation [64–66]. Chen et al. found that ALKBH3 can act as a demethylase for tRNA, removing the tRNA 1-methyladenosine (m1A) and 3-methylcytidine (m3C) position of tRNA [67]. Demethylated tRNAs were found to be more sensitive to ANG-mediated cleavage, and the resulting tsRNAs promoted cancer development by facilitating ribosomal assembly and inhibiting apoptosis [67]. Mechanistically, ALKBH3-induced tsRNAs enhance translation by directly binding 40s ribosomes [67]; meanwhile, tsRNAs are able to interact with cytochrome *c* (Cyt *c*), released from the mitochondria, thereby protecting cells from apoptosis (Fig. 2C) [67, 68]. Deletion of ALKBH1 (a m1A demethylase) [69] or ALKBH3 [67] restores tRNA m1A methylation levels, resulting in reduced tRNA cleavage and decreased tsRNA production.

Queuosine, a highly modified 7-deaza-guanosine, is present at the wobble anticodon of four amino acids (tRNA-His, tRNA-Tyr, tRNA-Asn, and tRNA-Asp) containing the 5’GUN anticodon [70]. In the presence of the Queuine tRNA-ribosyltransferase 1 /Queuine tRNA-ribosyltransferase 2 heterodimerase, queuosine is incorporated into the wobble anticodon 34 position of tRNA [71]. Wang et al. found that Q modification protected tRNA-His and tRNA-Asn from ribonuclease cleavage and suppressed tsRNA production [71]. In addition, 2′-O-methylation of the human elongator tRNA^Met^ (CAT) C34 wobble cytidine prevented site-specific cleavage of tRNA^Met^ (CAT) by stress-induced ANG and reduced tsRNA production [72]. Therefore, the chemical modification state of tRNA largely affects tsRNA production, however, it has not been established whether there are other factors that modulate tsRNA production.

## Action mechanisms of tsRNAs

### tsRNAs are directly involved in RNA silencing

**Fig. 3:**
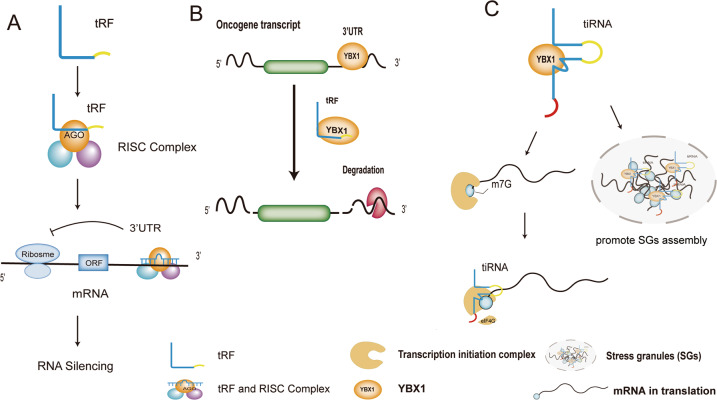
Action mechanisms of tsRNAs. **A** tRF binds AGO proteins to form the RISC complex. The complex performs a miRNA-like action, binding to the 3’ UTR of the target gene transcript through incomplete complementary and suppressing the expression levels of the gene. **B** tRF competes with the oncogene transcript to bind YBX1, resulting in the degradation of the oncogene transcript. **C** tiRNAs and YBX1 synergize to prevent eIF4G/A from initiating the translation and promote the SGs assembly.

tRNA derivatives exhibit miRNA-like effects [73]. tRFs can form complexes with Argonaute (AGO) proteins to interact with the 3’ untranslated regions (3’UTR) of target gene mRNAs and suppress the expressions of target genes (Fig. 3A) [74]. Maute et al. showed that the tRF-3, which was named CU1276 and derived from tRNA-Gly-GCC, had functional characteristics of miRNAs in B-cell lymphoma, including physical binding to AGO proteins and sequence-specific repression of mRNA transcription [75]. In addition, 5′tiRNAs in the cytoplasm interact with tRNase Z^L^ (tRNA endonuclease) as small guide RNA, guiding the 5’tiRNA-tRNase Z^L^ complex to specifically bind and cleave target genes, thereby downregulating the expression levels of the target genes [44, 76]. These findings suggest that tRFs and tiRNAs are directly involved in RNA silencing in a mechanism that is similar to that of miRNAs.

### tsRNAs indirectly modulate gene expressions via binding RNA-binding proteins

RNA-binding proteins (RBPs) are usually described as the proteins that interact with RNA via globular RNA-binding domains, thereby altering the fates of the interacting RNAs [77]. YBX1, an RBP belonging to the Y-box binding protein family, is abundantly expressed in many cancers [78–81]. Goodarzi et al. identified a novel category of tRFs derived from tRNA-Tyr, tRNA-Gly, tRNA-Glu, and tRNA-Asp. These tRFs can modulate the expressions of oncogenes via YBX1 and ultimately inhibit cancer metastasis [82]. Under hypoxia or other stress stimulation, more tRFs are produced in cells, which then compete with 3’UTR of oncogene transcripts to bind YBX1 (Fig. 3B). At this point, the YBX1 with oncogene mRNA complex is dissociated and the mRNA is destabilized. Thus, the expression levels of the oncogene are suppressed, which in turn inhibits cancer cell growth and metastasis [82]. In addition, Ivanov et al. revealed that tiRNAs and YBX1 synergized to prevent eIF4G/A from initiating translation (Fig. 3C) [59]. The damaged translation initiation induced the assembly of stress granules (SGs), and it was found that YBX1 was the only protein required for tiRNA-induced SG assembly [59]. Interestingly, in 2016, the same team reported that YBX1 could directly bind tiRNAs through its cold shock structural domain, which was necessary for the formation of SG, but was not necessary for the tiRNA-mediated translation inhibition [83].

### tsRNAs regulate translation

**Fig. 4:**
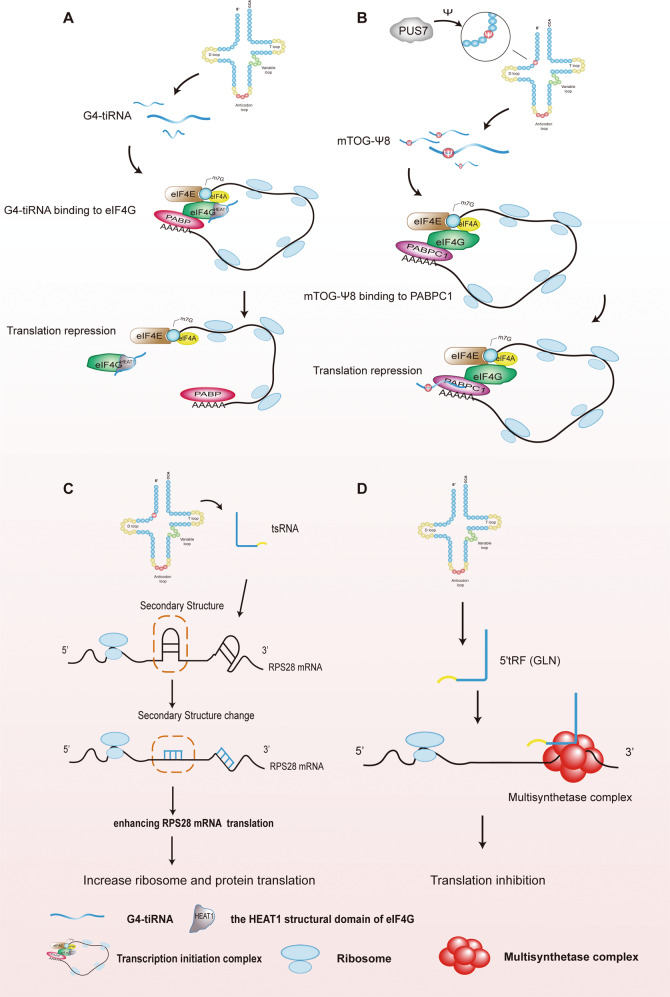
tsRNAs regulate translation. **A** The binding of G4-tiRNA to the HEAT1 domain of the translation initiation factor, eIF4G, inhibits translation initiation. **B** PUS7 modifies tRNA position 8 containing sequences of mTOGs to pseudouridine (Ψ), and this specific mTOG-Ψ8 is able to bind to the translation initiation factor, PABPC1, thereby inhibiting translation initiation. **C** tsRNA alters the secondary structures of RPS28 mRNA to enhance its translation and ultimately accelerates protein synthesis. **D** 5’tRF (GLN) inhibits the translation process by binding to the multisynthetase complex (MSC).

tiRNAs contain a segment of the terminal oligoguanine (TOG) motif at their 5′-terminal end [59], and the TOG motif confers tiRNAs translational repression activities by promoting the assembly of tetramer-containing G-quadruplex (G4) molecules [84, 85]. eIF4G, a crucial scaffolding protein, regulates the initiation of translation. Recently, Lyons et al. identified the tetrameric G4 form of TOG-containing tiRNAs (G4-tiRNAs), which was able to displace eIF4G from the m^7^GTP cap by interacting with the HEAT1 structural domain in eIF4G (Fig. 4A). This led to impaired scanning of the 40s ribosome on mRNAs and ultimately inhibited the initiation of translation [86].

A previous study documented that pseudouridylation synthase 7 (PUS7)-induced production of pseudouridine (Ψ) directed specific 5’tRFs to repress translation in stem cells [87]. These 5’tRFs were derived from tRNA-Ala, tRNA-Cys, and tRNA-Val and carried the TOG sequence at the 5’ end, which were named as mTOGs. They found that PUS7 modified the position 8 (U8) of mTOGs into a pseudopyrimidine (Ψ), hence termed the product as mTOG-Ψ8 [87]. Poly(A)-binding protein-1 (PABPC1), an important component of the translation initiation complex, interacts with translation initiation factors (eIF-4A/G and E) to form a closed-loop translation complex [87, 88]. PUS7-mediated mTOG-Ψ8 can bind PABPC1, leading to mTOG-dependent inhibition of the translation initiation complex (Fig. 4B) [87].

Successful translation cannot occur without ribosomes. Ribosomes consist of four ribosomal RNAs (rRNAs) and about 80 ribosomal proteins. Ribosomal protein S28 (RPS28) is a component of the 40s ribosomal subunit [89]. Kim et al. reported that Leu-CAG-tRNA-derived Leu-CAG 3′tsRNA can bind the coding sequence and 3′UTR of RPS28 mRNA, altering the secondary structure of RPS28 mRNA and thereby, enhancing its translation and maintaining RPS28 levels (Fig. 4C) [90]. An increase in ribosomal proteins enhances protein synthesis by the ribosomes, promoting cell growth and proliferation [90]. Sobala et al. identified a highly expressed 5’tRF (GLN) in HeLa cells, 19 nt in length, whose conserved “GG” nucleic acid sequence at the 3’ end confers itself translational repression activities without the need for complementation with target gene sequences [91]. It has been documented that 5’tRF (GLN19) can interact with the multisynthetase complex (MSC) to inhibit the translation process (Fig. 4D) [92]. However, the exact mechanisms remain to be further investigated.

## Roles in disease

### tsRNAs in immune responses

Evidence suggests that tsRNAs are involved in immune processes. Autoimmune diseases occur when the immune system fails to distinguish between self and foreign antigens, causing an immune response that results in damage to the body [93]. Systemic lupus erythematosus (SLE), a representative autoimmune disease, is associated with variable clinical features and complicated pathological mechanisms [94]. Xu et al. found that a total of 355 tsRNAs were differentially expressed in SLE, compared to normal controls. In GO and KEGG pathway analyses, tsRNAs and their related target genes were established to be enriched in immune response and immune system processes, as well as in signaling pathways such as Th1 and Th2 cell differentiation, T-cell receptor [95]. tRF-3009 from tRNA-Lue-TAA was first reported by Geng et al., who found that it was highly expressed in CD4 + T cells in SLE and positively correlated with SLE disease activity indices, lupus nephritis, and serum IFNα levels [96]. Mechanistically, tRF-3009 levels, in tandem with ATP and ROS levels, were elevated by IFNα treatment. Upon tRF-3009 knockdown, IFN-α-induced ROS and ATP were inhibited [96]. These findings imply that tRF-3009 may be involved in SLE development by regulating IFN-α-induced oxidative phosphorylation (OXPHOS) of CD4^+^ T cells [96]. However, the underlying mechanisms have not been elucidated.

Macrophages, including both polarized phenotypes of classically activated macrophages (M1) and selectively activated macrophages (M2), are involved in inflammatory processes and in immune responses [97]. Studies have shown that SLE patients initially have macrophage activation [98] and that M1 macrophages are associated with disease activity [99]. After isolating exosomes from bone marrow mesenchymal cells (MSC), Dou et al. found that MSC-exosome significantly inhibited the expression of M1 macrophage markers [100]. The expression levels of tsRNA-21109 were found to be significantly upregulated in the MSC group. Meanwhile, inhibition of tsRNA-21109 abolished the effects of MSC-exosomes on M1 macrophage polarization and elevated TNF-α and IL-1β levels in macrophages. Moreover, relative to healthy individuals, tsRNA-21109 levels were found to be significantly suppressed in SLE patients. These findings imply that tsRNA-21109 in MSC-exosomes might reduce SLE symptoms by inhibiting macrophage polarization towards the M1 phenotype [100].

### tsRNAs in metabolic disorders

**Fig. 5:**
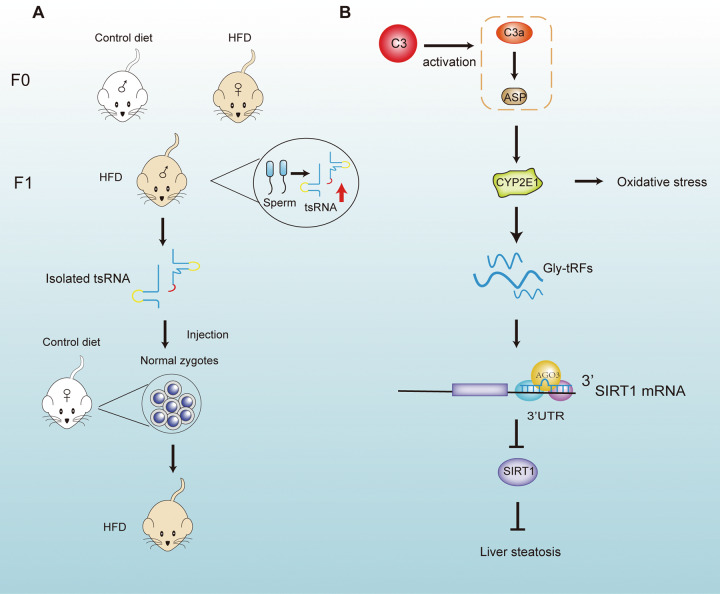
Representative mechanisms of tsRNAs in metabolic diseases. **A** Sperm tsRNA contributes to the intergenerational transmission of maternal HFD-induced addictive behaviors and phenotypes. **B** Complement C3 is involved in alcohol-induced liver damage and steatosis through the induction of Gly-tRFs.

Metabolic disorders are involved in the development of several diseases, including fatty liver, type 2 diabetes, and obesity, which threaten human health and life [101, 102]. Abnormalities in metabolic activities and pathways are increasingly associated with the dysregulation of sncRNAs [103, 104]. Indeed, as novel sncRNAs, tRF and tiRNA are also involved in different metabolic pathways. Sarker et al. identified that sperm tsRNAs could act as potential carriers to aid in the intergenerational transmission of addictive behaviors and obesogenic phenotypes induced by maternal high-fat diet (HFD) [105]. Maternal HFD induced the obesogenic phenotypes in the first generation (F1); the phenotypes in F1 could be replicated after injection of tRFs isolated from F1 male rat sperm into healthy fertilized individuals (Fig. 5A) [105]. Therefore, tRFs play a role in the intergenerational inheritance of metabolic disorders.

Wang et al. identified a small RNA referred to as tsRNA-06018, which was able to modulate adipogenesis in human bone marrow mesenchymal cells (HMSC) by targeting the 3’UTR of Stanniocalcin 2 (STC2) and then, the extracellular signal-regulated kinase 1/2 (ERK1/2) signaling pathway [106]. Inhibition of tsRNA-06018 disrupted the differentiation of HMSC to adipocytes [106]. Zhong et al. found that complement C3 could participate in alcohol-induced liver injury and steatosis via glycine tRNA-derived fragments (Gly-tRFs) (Fig. 5B) [107]. CYP2E1, a member of the cytochrome P450 mixed-function oxidase system, is involved in ethanol-induced oxidative stress in alcoholic fatty liver disease (AFLD) [107, 108]. C3 activation products (C3a and Asp) have been shown to promote Gly-tRF expression through CYP2E1 [107]. Moreover, C3-induced Gly-tRFs can interact with AGO3 to target the 3’UTR of SIRT1 and to inhibit SIRT1 expression [107]. The deletion of SIRT1, an NAD^+^-dependent deacetylase, enhances adipogenesis and impairs β-oxidation pathways [107, 109, 110]. Based on the above findings, it is plausible that tsRNAs play various roles in metabolic dysregulation, therefore, their impact on human health cannot be ignored.

### tsRNAs in malignancies

Frequent dysregulation of tsRNA in malignant tumors has been reported [32, 111–115]. In this section, we elucidate some representative functions and the underlying molecular mechanisms of tsRNA in cancers.

**Fig. 6:**
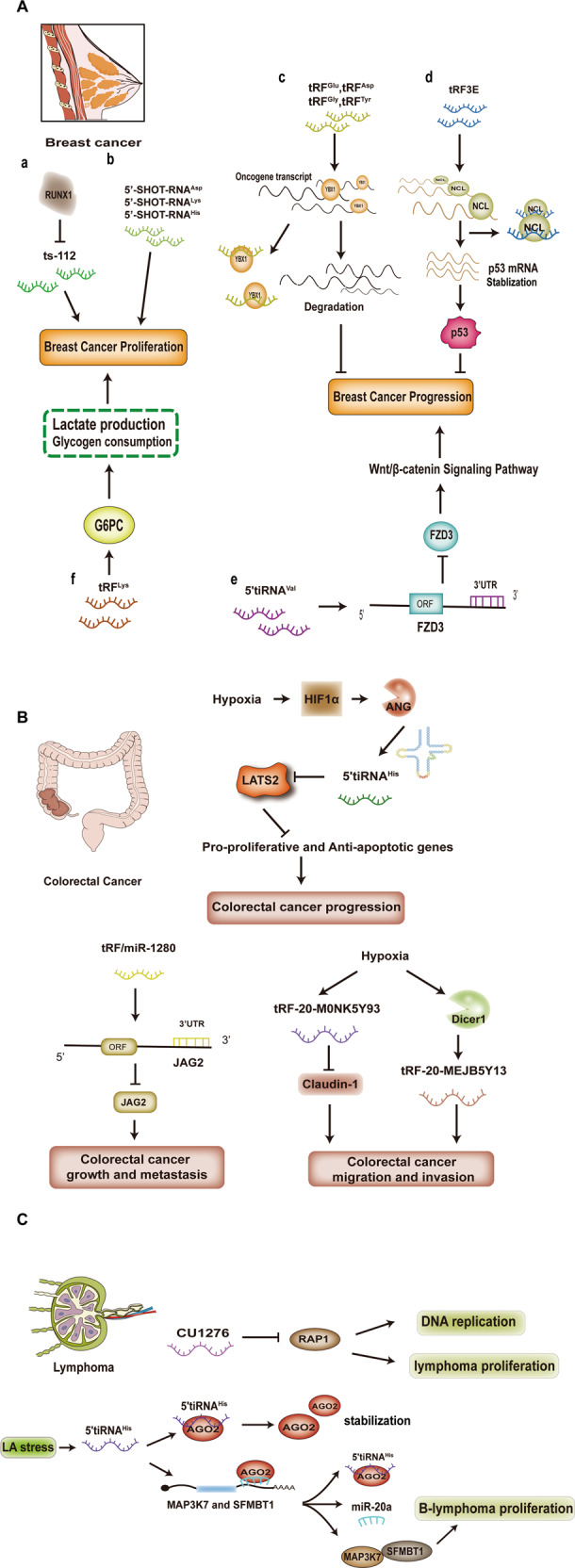
Representative mechanisms of tsRNAs in malignancies. **A** (**a**, **b**) ts-112 and 5’-SHOT-RNAs can promote breast cancer proliferation; **c** tRFs are able to compete with oncogene transcripts to bind YBX1, suppressing oncogene expression and inhibiting breast cancer progression; **d** tRF3E inhibits breast cancer progression by binding NCL and disrupting the inhibitory effect of NCL on p53 mRNA; **e** 5’-tiRNA-Val depresses the Wnt/β-catenin signaling pathway by targeting FZD3 to inhibit breast cancer progression; **f** tRF-Lys-CTT-010 promotes breast cancer cell proliferation by modulating the glucose metabolic pathway. **B** Hypoxia-induced 5’tiRNA-His-GTG targets LATS2, dysregulating the Hippo pathway and promoting the progression of colorectal cancer; tRF/miR-1280 inhibits colorectal cancer growth and metastasis by targeting the 3’UTR of the Notch ligand, JAG2 mRNA. Hypoxia-induced tRF-20-M0NK5Y93 inhibits the epithelial mesenchymal transition (EMT)-related molecule Claudin-1, and then suppresses colorectal cancer cell metastasis; Meanwhile, Dicer1-dependent expression of tRF-20-MEJB5Y13 was upregulated in response to hypoxic stimulation, leading to colorectal cancer migration and invasion. **C** CU1276 inhibits RAP1 to suppress lymphoma proliferation and to regulate DNA damage-induced molecular responses such as DNA replication; Lactate-induced 5’tiRNA binds AGO2 to maintain its stability. Accumulated 5’tiRNA competes with miR-20 to bind the AGO2 protein, resulting in increased expressions of the miR-20 target genes, SFMBT1 and MAP3K7, thereby promoting lymphoma proliferation.

Dysregulated expressions of tsRNAs have been reported in breast cancer [32, 82, 113, 114, 116]. Veronica et al. revealed that the tsRNAs were regulated by oncogenes and differentially expressed in different breast cancer stages. The expression levels of ts-29 gradually decreased from early to late stages of breast cancer, while ts-3 levels were dramatically downregulated in the late stages of breast cancer. In addition, they reported dysregulated ts-66 and ts-86 in breast cancer tissues, relative to the surrounding normal tissues [113]. Farina et al. identified four tsRNAs (ts-19, ts-29, ts-46, and ts-112) that selectively responded to the expression of the tumor suppressor RUNX1. In their study, ts-112, which has been shown to promote the proliferation of normal breast epithelium and breast cancer cells, was established to be significantly downregulated in RUNX1 overexpressed cells (Fig. 6A-a) [117]. Meanwhile, elevated tsRNAs levels were detected in breast cancer extracellular vehicles (EVs). A combination of the signatures of these tsRNAs with known miRNAs in tumors revealed that EVs specificity in cancer cells in circulation was greatly enhanced, distinguishing them from EVs of other cellular origins [118].

Estrogen receptor (ER) expression is closely associated with breast cancer development. The expression levels of 26 circulating tiRNAs from isoacceptors such as tRNA-Glu, tRNA-Lys, and tRNA-Gly in ER-positive tumors were found to be suppressed [114]; Honda et al. found that estrogen and its receptors enhanced the production of tiRNAs [32]. In breast cancer cell lines, expression levels of three tiRNAs, 5’-SHOT-RNA-Asp, 5’-SHOT-RNA-Lys, and 5’-SHOT-RNA-His, were markedly increased. Moreover, the effects of 5’-tiRNA-specific knockdown on cell proliferation have also been reported (Fig. 6A-b) [32]. Compared to non-inflammatory breast cancer, inflammatory breast cancer is associated with elevated tiRNA-Ala levels [114]. These findings suggest that tiRNAs are very likely to be involved in breast cancer progression [32].

Mechanistically, Goodarzi et al. identified novel tRFs, which had been induced by hypoxia stimulation. As earlier mentioned, these tRFs could suppress oncogene expression by occupying YBX1, thereby inhibiting breast cancer progression (Fig. 6A-c) [82]. Nucleolin (NCL) is an RBP that has been reported to be overexpressed in breast cancer [119]. Falconi M et al. established that a tRF3E, derived from tRNA-Glu, was able to interact with NCL to form a complex that disrupted the inhibitory effect of NCL on p53 mRNA translation and promoted p53 expression, which in turn regulated tumor growth (Fig. 6A-d) [119]. In addition, recently, Mo et al. reported that the tRNA-derived fragment 5’-tiRNA-Val inhibited the Wnt/β-catenin signaling pathway in breast cancer by directly targeting FZD3, thereby suppressing the malignant activities of breast cancer (Fig. 6A-e) [116]. Zhu et al. reported that tRF-Lys-CTT-010 can promote triple-negative breast cancer (TNBC) cell survival and proliferation. Mechanistically, tRF-Lys-CTT-010 regulates cell lactate production and glycogen depletion by targeting the catalytic subunit of glucose-6-phosphatase (G6PC) (Fig. 6A-f) [120].

Metastasis is the primary reason for colorectal cancer (CRC)-associated death [121, 122]. Li et al. reported that ANG levels are upregulated in CRC tissues [121]. The tiRNAs generated by ANG cleavage were abundantly expressed in CRC tissues and in highly metastatic cells, and played a role in ANG-mediated promotion of CRC metastasis [121]. Tao et al. established that half of the specific tRNA, 5’tiRNA-His-GTG, was upregulated in CRC tissues, and its production appeared to be a response to the hypoxic microenvironment in tumor cells. Its expressions were established to be regulated through the HIF1α/ANG axis (Fig. 6B) [28]. Moreover, 5’tiRNA-His-GTG enhanced the expressions of pro-proliferative and anti-apoptotic genes by targeting the LATS2 and“shutting down” the Hippo signaling pathway, thereby promoting CRC progression [28]. Previously, Huang et al. reported that a fragment, tRF/miR-1280, inhibited the Notch signaling pathway thus suppressing the cancer stem cell (CSC)-like cell phenotype in the CRC development process. Investigation of molecular mechanisms indicated that tRF/miR-1280 directly interacted with the 3’UTR of the Notch ligand, JAG2, thereby inhibiting CRC cell growth and metastasis (Fig. 6B) [123]. Recently, Luan et al. found that in CRC cells, the expressions of tRF-20-M0NK5Y93 under hypoxic conditions were suppressed, relative to control conditions [124]. tRF-20-M0NK5Y93 suppressed CRC cell transformation from epithelium to mesenchymal by targeting the epithelial mesenchymal transition (EMT)-related molecule (Claudin-1), thereby inhibiting CRC cell migration and invasion (Fig. 6B) [124]. In their other study regarding CRC metastasis, they reported that hypoxia-induced Dicer1 expression and the subsequent Dicer1-mediated upregulation of tRF-20-MEJB5Y13, leading to CRC cell migration and invasion (Fig. 6B) [125]. Wu et al. detected elevated plasma 5’-tRF-GlyGCC levels, relative to healthy controls. Moreover, its expressions in CRC cells and xenograft tissues were elevated, relative to their corresponding controls [126]. In CRC cells, the tRNA demethylase AlkB homolog 3 (ALKBH3) promotes 5’-tRF-GlyGCC levels [126]. The above findings form the basis for understanding the significance of tsRNA in colon cancer and clinical diagnosis.

B-cell lymphomas are solid tumors that involve B cells, and they mostly occur in immunocompromised and elderly patients. In a study of B-cell lymphomas, Maute et al. demonstrated that a tsRNA (CU1276) suppressed an important endogenous gene (RAP1) which is involved in many aspects of DNA dynamics, thereby inhibiting lymphoma proliferation and modulating DNA damage-induced molecular responses (Fig. 6C) [75]. Mo et al. found that in an Epstein–Barr virus (EBV)-immortalized B lymphoblastic cell line (LCL) model, lactate (LA) induced three 5’-tiRNAs (5’-HisGUG, 5’-ValAAC, and 5’-GlyGCC) generation, which promoted B-cell lymphoma proliferation via 5’tiRNA-His-GUG [127]. Mechanistically, LA-induced 5’tiRNA-His-GUG selectively bound the chromatin regulator, argonaute-2 (AGO2), to maintain AGO2 protein stability. Under LA stress stimulation, 5’tiRNA-His-GUG accumulated and competed with miR-20a to release MAP3K7 and SFMBT1 expression, for the convenience of B-lymphoma cell survival and proliferation (Fig. 6C) [127]. Meanwhile, the expression levels of 5’tiRNA-His-GUG in PBMCs of B-cell lymphoma patients were found to be closely associated with lactate dehydrogenase (LDH; an indicator of lactate in plasma) [127]. These findings imply that 5’tiRNA-His-GUG is a potential diagnostic and therapeutic molecular target.

Drug resistance is highly correlated with cancer treatment failure. Hypoxia enhanced the production of tRNA-derived fragments (tDR-0009 and tDR-7336), which were remarkably elevated in TNBC cells [128]. Further analyses revealed that the two elevated tDRs are involved in the maintenance of stem cell numbers and interleukin (IL)‐6 responses [128]. IL-6 is involved in multidrug resistance by activating JAK/STAT3, PI3K/Akt, and other pathways [129]. Moreover, protein–protein interaction (PPI) analysis showed that tDR-0009/tDR-7336 is closely associated with the STAT3 protein [128]. STAT3, the downstream of IL-6, can trigger the NF-κB signaling pathway by upregulating the expression of tumor necrosis factor receptor superfamily member 1A (TNFRSF1A) [130]. The activation of STAT3/NF-κB signaling can lead to chemotherapy resistance in TNBC [131]. Therefore, tDR-0009/tDR-7336 is likely to affect hypoxia‐induced chemoresistance by regulating IL-6/STAT3 signaling in TNBC [128].

Yang et al. investigated the significance of tsRNA in cancer drug resistance. In prostate cancer (PCa) cells, cisplatin treatment induced ANG-dependent tRF-315 production [132]. tRF-315 suppressed cisplatin-mediated apoptosis and mitochondrial dysfunction [132]. Moreover, tRF-315 affected the cell cycle altered by cisplatin by targeting GADD45A, a tumor suppressor gene. As a result, tRF-315 protected PCa cells against cisplatin-induced apoptosis [132]. tRF-315 inhibitors might have important applications on the clinical improvement of cisplatin resistance [132]. Therefore, tsRNA is a potential therapeutic target for overcoming cancer drug resistance.

## Clinical applications and detection methods

Extracellular RNA biomarkers, which are non-invasive and can be stably present in body fluids, have attracted great interest from researchers. In particular, tRFs and tiRNAs, whose potential as biomarkers in clinical applications is gradually emerging. tRFs and tiRNAs are abundantly present in serum or saliva [133, 134]. Wang et al. identified six tRFs from the 5’ end of tRNAs that were markedly decreased in plasma samples from patients with early breast cancer (EBC) [115]. Suppressed tRF-Glu-CTC-003 levels in HER2 + EBC patients were correlated with poorer overall survival and disease-free survival outcomes [115]. These tRFs are potential diagnostic biomarkers for EBC. In addition, tsRNAs can be selectively exported into Evs, and exosome-carried tsRNAs have been used as biomarkers for clinical diagnostic applications [36, 135]. Zhao et al. found that androgen-dependent tiRNAs were upregulated in PCa patients [136]. Elevated levels of these tiRNAs were strongly correlated with poorer clinicopathological parameters and shorter biochemical recurrence times. 5’-tRNA-Glu-CUC hemicycle levels in serum were established to be higher in metastatic castration-resistant PCa patients, relative to patients with limited PCa, indicating that these tsRNAs have the potential to be circulating biomarkers and prognostic predictors for PCa [136]. Zhu et al. reported significantly higher levels of tsRNA-ValTAC-3, tsRNA-GlyTCC-5, tsRNA-ValAAC-5, and tsRNA-GluCTC-5 in plasma exosomes of HCC patients, compared to healthy controls, suggesting that these exosomal tsRNAs are potential “liquid biopsy” tumor diagnostic biomarkers [137].

To detect and study tsRNAs, researchers have established several tsRNA databases for communication and research (supplementary table). tRFexplorer (https://trfexplorer.cloud/), researchers can forecast and analyze the potential biological effects of tRFs [138]. MINTbase v2.0 (http://cm.jefferson.edu/MINTbase/), which includes the information about mitochondrial and nuclear tRFs in a wide range of human tissue samples, provides information on tRF expression, its specific data and its parent tRNAs [48]. Mitochondrial and nuclear tRF mapping (MINTmap; https://github.com/TJU-CMC-Org/MINTmap/), which helps to distinguish tRFs from short RNA-seq databases in a fast and deep manner and provides raw as well as normalized abundance data of tRFs [139]. BBCancer (http://bbcancer.renlab.org/) contains a database of expression of six classes of RNA types, including tRNA-derived fragments, in blood samples of normal individuals and of patients with different cancer types [140]. OncotRF (http://bioinformatics.zju.edu.cn/OncotRF) involves qualitative and quantitative analyses of aberrantly expressed tRFs in 33 cancer types, and is able to analyze abnormally expressed tRFs as well as gene correlations, tRF-related functional enrichment and survival analyses [141]. TsRBase (http://www.tsrbase.org.) contains information related to 121,942 tsRNAs in 20 species. This database provides researchers with validated tsRNA-related functions as well as target gene-binding sites and collects the latest literature on tsRNAs, which helps researchers to understand cutting-edge information in this field [142]. These excellent and comprehensive resources offer researchers with the possibility of exploring the expressions and molecular mechanisms of tsRNA in diseases.

## Challenges and outlook

We reviewed the biogenesis and functions of tsRNA and discussed the possible molecular mechanisms involved in immunological diseases, metabolic disorders and malignant tumors. However, due to technical limitations for discovering and confirming tsRNA, studies on tsRNA are still in early stages. Several crucial issues deserve in-depth attention.

First, how is tsRNA degraded? We reviewed the origin, formation processes and important functions of tsRNAs in biological processes. However, as a functional molecule, it is important to determine where it ends up. Which molecules are involved in tsRNA degradation and what are the molecular mechanisms behind it? It is also important to determine whether tsRNA dysregulation is associated with abnormal degradation processes, and whether it can directly or indirectly affect disease development. A cullin-RING ubiquitin ligase, containing the substrate adapter, ZSWIM8, is responsible for target-directed microRNA degradation [143]. This complex target and ubiquitinate the microRNA-bound AGO protein, and finally promote AGO degradation and the subsequent microRNA degradation [143]. The biological characteristics of tsRNA are comparable to those of microRNAs. Therefore, we postulate that the degradation mechanisms of some tsRNAs may also be associated with the above ubiquitin complex.

Second, in the course of its functions, does tsRNA interact with other noncoding RNAs? Interactions between tsRNA and microRNA have been reported. tsRNA can prevent microRNA from binding its target gene by interacting with microRNA, to relieve the inhibition of target gene expressions [127]. In addition, lncRNA and circRNA sponge microRNA to disrupt the inhibition of microRNA on target genes [144, 145]. However, it has not been established whether lncRNAs and circRNAs can interact with tsRNA to affect its biological functions. Moreover, there is a need to assess the intricate molecular interaction networks between noncoding RNAs, such as piRNA, microRNA, circRNA, tsRNA, and lncRNA that coordinate or restrict each other in the course of disease development.

Third, what are tsRNA-associated chemical modifications, and can these modifications affect their biological functions? Like tRNA, there are chemical modifications on tsRNA, however, the types and effects of these modifications remain to be revealed. A recent study showed aminoacylation modifications on leu-CAG 3’tsRNA from tRNALeu. When aminoacylation is inhibited, leu-CAG 3’tsRNA levels are suppressed, affecting its biological functions of promoting cell proliferation and inhibiting cell apoptosis [146]. This indicates that chemical modifications on tsRNA are likely to be closely associated with its biological functions. However, whether there exists other kind modification of tsRNA and its potential role are waiting for us to further study and explore.

In addition, the portability and accuracy of tsRNA as biomarkers for clinical diagnosis and prognosis should be confirmed. Synergistic applications of tsRNA inhibitors or mimics with anticancer drugs should be investigated. Moreover, the potential clinical values of tsRNAs combined with bioengineered materials such as nano-biomaterials should be evaluated. All of these unknowns guide us to further explore and increasingly advanced technology will help us unmask these mysteries.

In conclusion, increasingly, new tsRNAs are being identified, and their roles as well as underlying mechanisms in diseases continuously clarified. In-depth studies of tsRNA will elucidate disease occurrence and development. Moreover, in future, tRNA derivatives will have important clinical values as new diagnostic biomarkers and therapeutic targets.

## Supplementary information

supplementary table legend

supplementary table

abbreviation list

## Data Availability

All data generated or analyzed during this study are included in this published article and supplementary materials.
