# Supplementary material for: Deciphering the tRNA-derived small RNAs: origin, development, and future
Source: Cell Death Dis. 2021 Dec 21;13(1):24. doi: 10.1038/s41419-021-04472-3 (PMC8692627; doi:10.1038/s41419-021-04472-3)
Supplement: Supplementary file 1 — supplementary table legend [file 41419_2021_4472_MOESM1_ESM.docx]

**Supplementary table legend**

The resource databases that are currently publicly available for analyzing tsRNAs.
