# Supplementary material for: Deciphering the tRNA-derived small RNAs: origin, development, and future
Source: Cell Death Dis. 2021 Dec 21;13(1):24. doi: 10.1038/s41419-021-04472-3 (PMC8692627; doi:10.1038/s41419-021-04472-3)
Supplement: Supplementary file 2 — supplementary table [file 41419_2021_4472_MOESM2_ESM.pdf]

**tsRNA databases database**	**Note**
tRFexplorer[140]	Contains tsRNA expression profiles in all cell lines in NCI-60 as well as in all TCGA tumor types
MINTbase v2.0[48]	Comprehensive tRF database containing nuclear and mitochondrial fragments of different cancer gene maps
MINTmap[141]	Rapid and accurate analysis and quantification of tRFs in RNA-Seq databases
BBCancer[142]	It is an open database providing the expression of 6 types of RNA in blood samples from patients with various cancer types
OncotRF[143]	A comprehensive database exploring the function of tRFs in human cancers and identifying diagnostic and prognostic biomarkers of cancer
TsRBase[144]	A comprehensive database for querying tsRNA expression and function in a wide range of species

URL link

https://trfexplorer.cloud/

http://cm.jefferson.edu/MINTbase/

https://github.com/TJU-CMC-Org/MINTmap/

http://bbcancer.renlab.org/

http://bioinformatics.zju.edu.cn/OncotRF

http://www.tsrbase.org.
