# Supplementary material for: Deciphering the tRNA-derived small RNAs: origin, development, and future
Source: Cell Death Dis. 2021 Dec 21;13(1):24. doi: 10.1038/s41419-021-04472-3 (PMC8692627; doi:10.1038/s41419-021-04472-3)
Supplement: Supplementary file 3 — abbreviation list [file 41419_2021_4472_MOESM3_ESM.pdf]

**Full name**	**abbreviation**
tumor necrosis factor-α	TNF-α
tRNA-derived small RNAs	tsRNAs/tDR
tRNA-derived fragments	tRFs
tRNA halves/tRNA-derived stress-inducible RNAs	tiRNAs
triple negative breast cancer	TNBC
transfer RNA	tRNA
the Janus kinase	JAK
terminal oligoguanine	TOG
Systemic lupus erythematosus	SLE
stress granules	SGs
snoRNAs-derived fragments	sdRNAs
small nucleolar RNAs	snoRNAs
small nuclear RNAs	snRNAs
small non-coding RNAs	SncRNAs
small interfering RNAs	siRNAs
extracellular signal-regulated kinase 1/2	ERK1/2
signal transducer and activator of transcription 3	STAT3
sex hormone-dependent tRNA-derived RNAs	SHOT-RNAs
Protein Kinase B	Akt
RNA binding proteins	RBPs
rRNAs-derived RNA fragments	rRFs
ribosomal RNAs	rRNAs
Ribosomal protein S28	RPS28
prostate cancer	PCa
precursor tRNAs	pre-tRNAs
polynucleotide phosphorylase	PNPASE
Poly(A)-binding protein-1	PABPC1
PIWI-interacting RNAs	piRNAs
phosphotidylinositol 3 kinase	PI3K
oxidative phosphorylation	OXPHOS
nucleotide	nt
Nucleolin	NCL
nuclear transcription factor-kappa B	NF-kB
non-coding RNAs	ncRNAs
microRNAs	miRNAs
long noncoding RNAs	lncRNAs
large tumor suppressor kinase 2	LATS2
lactate dehydrogenase	LDH
lactate	LA
interleukin 6	IL-6
interleukin 1 beta	IL-1β
high-fat diet	HFD
glucose-6-phosphatase	G6PC
extracellular vesicles	EVs
Estrogen receptor	ER
epithelial mesenchymal transition	EMT

endonuclease Z	RNaseZ
endonuclease P	RNaseP
early breast cancer	EBC
cytoplasmic homologous ribonuclease Z2	ELAC2
cytochrome c	Cytc
colorectal cancer	CRC
circular RNAs	circRNAs
bone marrow mesenchymal cells	MSC
Argonaute	AGO
angiopoietin	ANG
AlkB homolog 3	ALKBH3
alcoholic fatty liver disease	AFLD
3' untranslated region	3’UTR
cancer stem cell	CSC
tumor necrosis factor receptor superfamily member 1	TNFRSF1A
